# The Footprint of the Inter-decadal Pacific Oscillation in Indian Ocean Sea Surface Temperatures

**DOI:** 10.1038/srep21251

**Published:** 2016-02-17

**Authors:** Lu Dong, Tianjun Zhou, Aiguo Dai, Fengfei Song, Bo Wu, Xiaolong Chen

**Affiliations:** 1 State Key Laboratory of Numerical Modeling for Atmospheric Sciences and Geophysical Fluid Dynamics, Institute of Atmospheric Physics, Chinese Academy of Sciences, Beijing 100029, China; 2 University of Chinese Academy of Sciences, Beijing 100049, China; 3 Joint Center for Global Change Studies (JCGCS), Beijing 100875, China; 4 Department of Atmospheric and Environmental Sciences, University at Albany, SUNY, Albany, New York, USA; 5 National Center for Atmospheric Research (NCAR), Boulder, CO, USA

## Abstract

Superimposed on a pronounced warming trend, the Indian Ocean (IO) sea surface temperatures (SSTs) also show considerable decadal variations that can cause regional climate oscillations around the IO. However, the mechanisms of the IO decadal variability remain unclear. Here we perform numerical experiments using a state-of-the-art, fully coupled climate model in which the external forcings with or without the observed SSTs in the tropical eastern Pacific Ocean (TEP) are applied for 1871–2012. Both the observed timing and magnitude of the IO decadal variations are well reproduced in those experiments with the TEP SSTs prescribed to observations. Although the external forcings account for most of the warming trend, the decadal variability in IO SSTs is dominated by internal variability that is induced by the TEP SSTs, especially the Inter-decadal Pacific Oscillation (IPO). The IPO weakens (enhances) the warming of the external forcings by about 50% over the IO during IPO’s cold (warm) phase, which contributes about 10% to the recent global warming hiatus since 1999. The decadal variability in IO SSTs is modulated by the IPO-induced atmospheric adjustment through changing surface heat fluxes, sea surface height and thermocline depth.

The regions around the Indian Ocean (IO) are home to about one third of world’s population, with the majority living in developing countries that are highly vulnerable to climate variability and change. Considerable evidence^1^,^2^,^3^,^4^,^5^ shows that sea surface temperature (SST) variations in the IO have large impacts on rainfall and atmospheric circulation over Africa and South Asia, especially on the South and East Asian monsoons. The IO has experienced a significant warming trend during the twentieth century^6^,^7^,^8^, which has been examined and attributed to human activity^9^,^10^. However, pronounced decadal (including multi-decadal) fluctuations are also present in IO SSTs, overlying the warming trend^11^. A basin-wide pattern dominates the decadal variability in IO SSTs^12^. Recent modeling studies^13^,^14^,^15^ have focused on IO sea level decadal variability; and local wind forcing has been proposed as the main driver, with remote forcing also having significant contributions from the Pacific transmitted through the Indonesian throughflow. There are, however, few studies on the cause of the decadal SST variability in the IO, even though many mechanisms have been proposed to explain the decadal SST variability in the Pacific and Atlantic Oceans^16^,^17^,^18^. Many studies have shown that the IO and Pacific Ocean are closely linked on inter-annual to multi-year time scales, and the tropical IO can respond to remote forcing from the Pacific El Niño and La Niña^19^,^20^,^21^. However, few studies have examined their relationship on decadal time scales^22^. Improved understanding of IO decadal SST variability can help understand and predict decadal climate variations in and around the IO. This study aims to reveal the mechanisms responsible for the IO decadal variability. We use model experiments, observations and reanalysis datasets to identify remote influences from the Pacific Ocean on the IO. The details of the model experiments and analysis method are provided in Method section.

## Results

**Figure 1:**
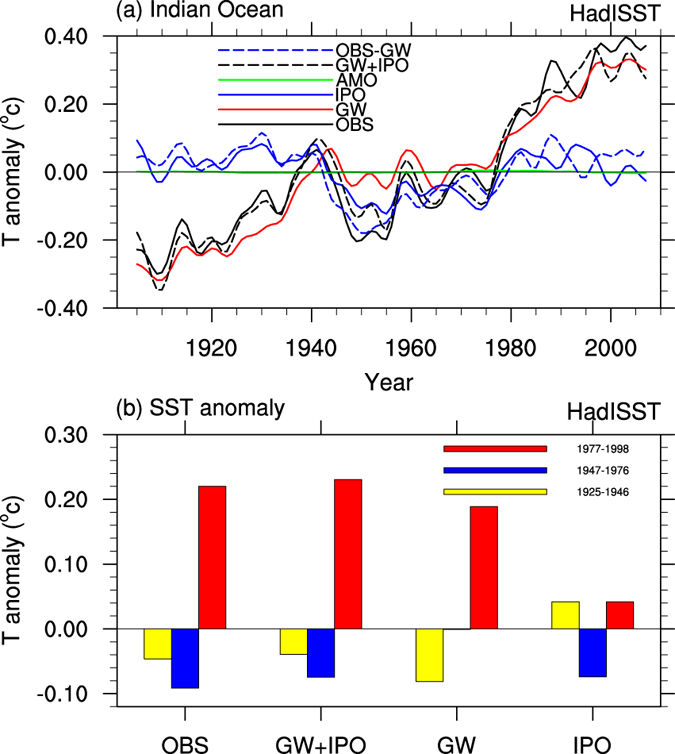
Contributions of the global warming, IPO and AMO to IO SSTs in observations. (**a**) Time series of the 8-year low-pass Lanczos filtered annual SST anomalies averaged over the Indian Ocean (20°S–20°N, 40°–120°E) from the HadISST dataset (black solid line) and the components represented by the global warming (GW, red), IPO (blue solid) and AMO (green) modes, the GW plus IPO modes (black dashed), and the observed minus the GW mode (blue dashed). (**b**) Epoch mean of the Indian Ocean SST anomalies based on the time series in (**a**) during the warm IPO phase (1925–1946, yellow bars), the cold IPO phase (1947–1976, blue bars), and the warm IPO phase (1977–1998, red bars). This plot was created by NCAR Command Language^43^.

To quantify the effects on IO SSTs, we first compare the SST anomalies represented by the global warming (GW), Inter-decadal Pacific Oscillation (IPO) and Atlantic Multi-decadal Oscillation (AMO) modes during 1901–2011 (Supplementary Fig. S1)^23^. The IO-averaged SST anomaly time series from the HadISST dataset^24^ is largely represented by the GW mode (correlation coefficient r = 0.93, red line in Fig. 1a). Combining the GW and IPO modes increases the agreement with the observations (r = 0.97, black dashed line in Fig. 1a). Removing the GW mode from the observed SST yields mostly decadal to multi-decadal variations that closely follow the IPO-induced SST variations (r = 0.79, blue lines in Fig. 1a). These results suggest that the IPO plays an important role in controlling the decadal variability of the IO SSTs. The warm (cold) phase of the IPO favors a warm (cold) SST anomaly (up to 0.08 ^o^C) in the IO (Fig. 1b). Thus, the decadal variability of the IO SST is linked to the IPO.

We realize that the GW mode obtained here may partly contain the decadal variations induced by the IPO (e.g., after about year 2000) and other internal variability^25^; however, removal of this decadal component from the GW mode should further reduce the correlation between the detrended SST and the GW mode and increase the importance of the IPO mode for the decadal variability in the IO, where the AMO has no effect (Fig. 1). The large volcanic cooling after 1982 and 1991 also degrades the relationship between the IPO-induced and OBS-GW lines in Fig. 1a after the 1970s.

To quantify the relative importance of different factors in the IO decadal variability, we conducted a suite of experiments using the Community Earth System Model version 1.2 (CESM1.2)^26^ forced with observed historical forcings and with or without prescribed SST anomalies from observations^24^ in the tropical eastern Pacific (TEP)–the tropical lobe of the IPO domain (Supplementary Table S1 and S2). The external forcings, which cover 1850–2012 or a shorter period of 1950–2012 (Supplementary Table S1), are based on the Coupled Model Intercomparison Project phase 5 (CMIP5) forcing agents^27^. Our dynamic-model-based attribution has a distinct advantage over the empirical approach above (Fig. 1) in that it can isolate the effects of the IPO more clearly.

The IO-averaged annual-mean SST increases in response to increased radiative forcing (Supplementary Fig. S2a). The simulated and observed IO SSTs are significantly correlated (r = 0.83 for one member of the *All forcing* + *TEP SST* and r = 0.67 for *All forcing* runs). These correlations are largely due to the long-term warming trend. Once linearly detrended, the *All forcing* + *TEP SST* run still follows the observed variations closely (r = 0.62); however, the correlation decreases to 0.28 for the *All forcing* runs, although it is still significant (Supplementary Fig. S2b).

**Figure 2:**
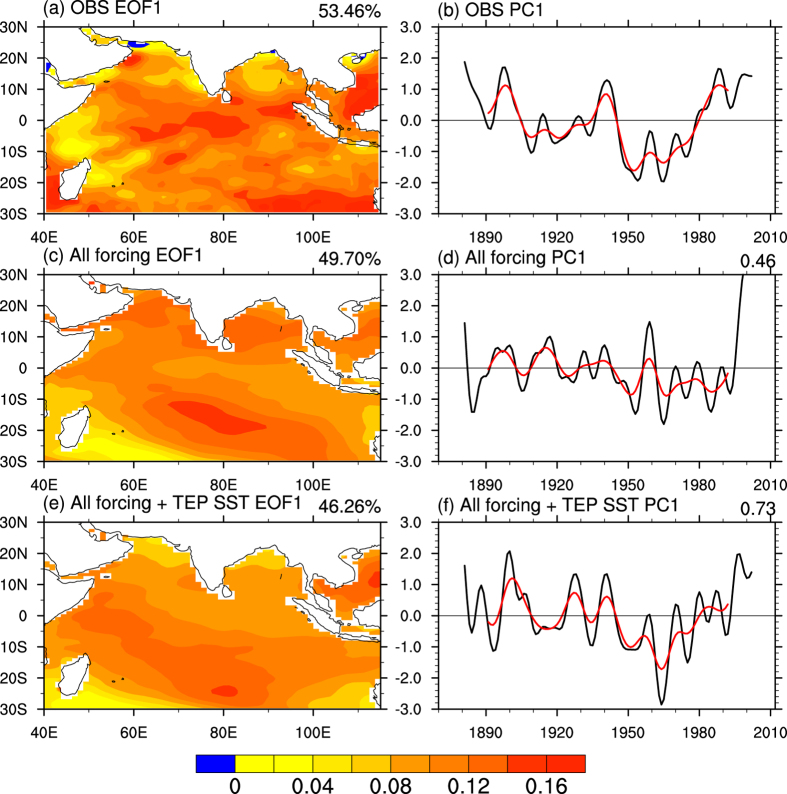
The dominant mode of Indian Ocean decadal variability. The leading EOF pattern and associated standardized principal component (PC, black lines) of the 8-year low-pass filtered annual SST in the Indian Ocean from (**a**,**b**) HadISST, (**c**,**d**) one *All forcing* run, and (**e**,**f**) one *All forcing* + *TEP SST* run during 1871–2012. The SST data were linearly detrended prior to the EOF analysis. The values given on the top-right of the panels represent the explained percentage variance by this mode in (**a**,**c**,**e**), and the correlation coefficients with the HadISST PC in (**d**,**f**). The red lines show the 15-year low-passed filtered time series of black lines. This plot was created by NCAR Command Language^43^.

The first leading mode of the linearly detrended SST in the IO from HadISST features a basin-wide pattern with predominant decadal variations (Fig. 2a,b). Despite capturing the basin-wide pattern, the detrended *All forcing* run with the fully coupled ocean and atmosphere fails to reproduce the observed temporal evolution (Fig. 2c,d), as one would expect since the temporal evolution is realization-dependent. In contrast, the experiment with TEP SST prescribed (*All forcing* + *TEP SST*) remarkably reproduces the observed IO decadal evolution (r = 0.73, Fig. 2e,f), with the phase transitions around the 1920s, 1940s, and 1970s in the observations being captured by the *All forcing* + *TEP SST* run (Fig. 2f). These results suggest that the IO decadal variability is modulated by the well-known decadal variability in TEP SSTs (i.e., IPO)^16^. This conclusion is consistent with the observational result shown in Fig. 1, and is further supported by the results from additional model runs. These runs include another single *All forcing* + *TEP SST* run, *piControl* + *TEP SST* run and three-member ensemble of the *All forcing* + *TEP SST* runs during 1950–2012 (Supplementary Table S1), which all successfully reproduce the observed phase transition in the late 1970s (Supplementary Fig. S3). The timing of this transition is consistent with the well-known phase transition of the IPO^28^. In the *piControl* + *TEP SST* run, there was no external forced signal in IO SSTs, and the experiment only reflects the influences of the observed TEP SST and local internal variability on IO SSTs. In the three-member ensemble mean of the *All forcing* + *TEP SST* runs, the uncorrelated local internal variability is reduced by the ensemble averaging, and the result mainly represents the effects of both the external forcing and the TEP SSTs. Note that the mode of *piControl* + *TEP SST* run shows a stronger east-west asymmetry and higher correlation with IPO than others (Supplementary Fig. S3c and S3d), which is due to the absence of the effect of external forcing in this run. These experiments reveal that, as long as the observed SST in the TEP is prescribed in the model, the experiments can capture the phase transition of the IO decadal mode during the late 1970s, regardless whether the external forcing or local internal variability is considered. Thus, these results further demonstrate that the phase transition of the decadal mode in IO SSTs arises from the remote influence of the IPO from the TEP.

To clarify the relative contributions of external forcing versus internal variability, and to which extent the remote TEP SSTs affect the IO decadal variability, we examine the SST differences between the warm and cold phases of the IPO. In the observations, the impact of the TEP SST is overwhelmed by the influence of the external forcing, resulting in a decadal warming from 1951–1976 to 1977–1998 (Supplementary Fig. S4a) and from 1977–1998 to 1999–2012 (Supplementary Fig. S5a). When the impact of external forcing is removed using observation minus ensemble mean of the *All forcing* runs, the warm (cold) phase of the IPO is associated with a warmer (colder) IO by up to 0.4 °C (Supplementary Fig. S4c and S5c). Such relationship can be caused solely by the TEP SST forcing (Supplementary Fig. S4e and S5e), and even by the internal variability component of the TEP SST (Supplementary Fig. S4f and S5f), i.e., with the impact of the external forcing being subtracted out. Thus, the decadal warming (cooling) in TEP SSTs induces a decadal warming (cooling) over most of the IO, and this is mainly due to the IPO variability.

**Figure 3:**
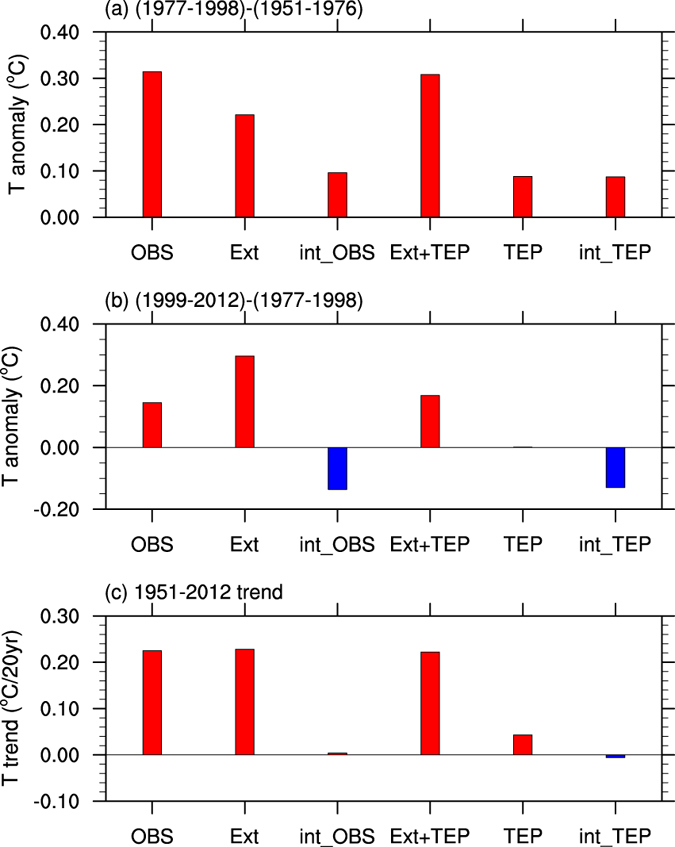
Contributions of different factors to Indian Ocean decadal and long-term changes. (**a**) Indian Ocean (20°S–20°N, 40°E–120°E) averaged SST decadal changes (^o^C) from 1951–1976 (an IPO cold phase) to 1977–1998 (an IPO warm phase) based on HadISST (OBS), the ensemble mean of the three *All forcing* runs (Ext), the internal variability in HadISST (int_OBS) obtained using OBS minus Ext, the ensemble mean of the three *All forcing* + *TEP SST* runs (Ext_TEP), one *piControl* + *TEP SST* run (TEP), and the effect of internal TEP SST (int_TEP) obtained using Ext_TEP minus Ext. Panel (**b**) is the same as (**a**), but for the difference between the IPO cold phase from 1999–2012 and the IPO warm phase from 1977–1998. (**c**) The Indian Ocean averaged SSTs linear trend (^o^C per 20 years) during 1951–2012. This plot was created by NCAR Command Language^43^.

To quantitatively compare their relative contributions, the IO-averaged changes over the different IPO phase periods are calculated (Fig. 3). The IO is about 0.31 ^o^C warmer during 1977–1998 (an IPO warm phase) than during 1951–1976 (an IPO cold phase) (Fig. 3a) based on HadISST. External forcing (based on the *All forcing* runs) accounts for ~ 71% of this decadal warming (Ext bar in Fig. 3a), with the residual being attributed to internal variability (int_OBS bar in Fig. 3a). For the decadal difference between 1999–2012 (an IPO cold phase) and 1977–1998 (an IPO warm phase) (Fig. 3b), the IO again exhibits a warm anomaly of 0.15 ^o^C (OBS bar). External forcing has a positive contribution of 0.30 ^o^C, implying a contribution of − 0.15 ^o^C by internal variability. Therefore, internal variability enhances (weakens) the warming effect of the external forcing over the IO by about 50% during the IPO warm (cold) phases. With the observed TEP SSTs included in the model (Ext + TEP bar in Fig. 3a,b), the magnitude of the decadal changes is almost the same as that derived from the observations, again confirming the key role of the IPO in reproducing the decadal change in IO SSTs and implying a dominant role of the IPO for the internal decadal variability in IO SSTs. Internal variability of the TEP SST (int_TEP bar) is responsible for the internal component of the observations (int_OBS bar). For the long-term linear trend during 1951–2012 (Fig. 3c), which includes almost a full cycle of the IPO and thus it has little effect on the trend, the warming trend is fully attributable to the external forcing, with little effect from the internal variability. In summary, the external forcing accounts for most of the IO warming trend, while internal variability associated with the remote TEP SSTs (mainly IPO) dominates the decadal variations in IO SSTs.

**Figure 4:**
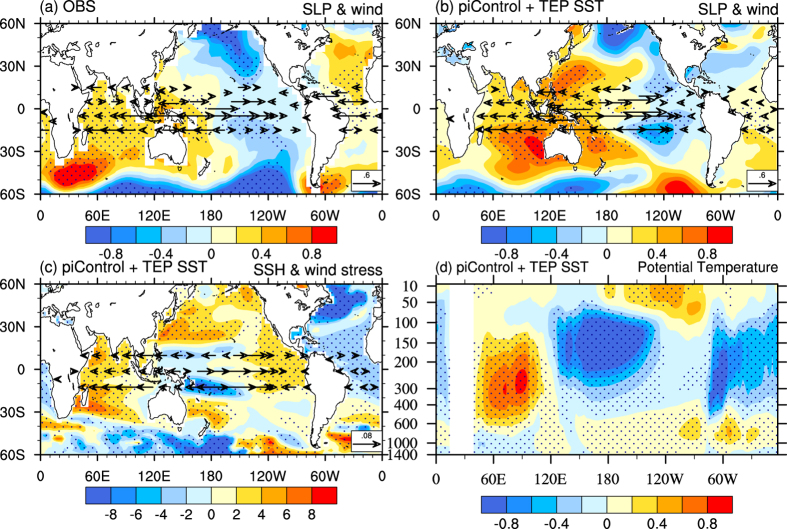
The mechanism of the IPO remote influence on Indian Ocean decadal variability. Decadal difference patterns between the IPO warm phase from 1977–1998 and the IPO cold phase from 1951–1976 (i.e., 1977–1998 minus 1951–1976) for (**a**) sea level pressure (hPa) from HadSLP2 and surface zonal wind vectors (m/s) from JRA-55 reanalysis, (**b**) same as (**a**) but from one *piControl* + *TEP SST* run, (**c**) sea surface height (cm) and zonal wind stress vectors (dyne/cm^2^) from the *piControl* + *TEP SST* run, and (**d**) ocean potential temperature (°C) averaged over 15°N–15°S from the *piControl* + *TEP SST* run. Note that the SLP in (**a**) is 1977–1998 minus 1958–1976 due to the time limitation of the HadSLP2 dataset. The dotted areas are statistically significant at the 5% level based on a Student’s *t*-test. This plot was created by NCAR Command Language^43^.

Compared with the IPO cold phase from 1951–1976, during the IPO warm phase from 1977–1998 there are a trans-basin see-saw pattern in sea level pressure (SLP) anomaly and weakened trade winds in the tropical Pacific Ocean, with positive SLP anomalies over the entire IO, which are seen in both observations and atmospheric reanalysis (Fig. 4a and Supplementary Fig. S6a). External forcing does not result in a clear trans-basin SLP see-saw pattern or significant anomalous winds in the tropical Pacific (Supplementary Fig. S6b). Adding the observed TEP SST variations to the model, the decadal change patterns in SLP and trade winds are qualitatively reproduced (cf. Fig. 4a and Supplementary Fig. S6c). This demonstrates that the TEP SSTs play a more important role than the external forcing in changing the Pacific trade winds and SLP. This is further illustrated by the case in which external forcing was set to the pre-industrial level, the change patterns (especially for SLP) forced by TEP SSTs alone (Fig. 4b) are in general agreement with the observations and reanalysis (Fig. 4a).

The positive SLP anomalies over the IO and the tropical western Pacific during the IPO warm phase imply an anomalous descending motion over these regions, which is consistent with a weakened Walker circulation as implied by the surface wind anomalies. Reduced cloudiness occurs over the IO (Supplementary Fig. S7a), which in turn generates increased surface net energy flux into the IO, although it is not uniform (Supplementary Fig. S7b). We note that the cloudiness is increased in the global atmospheric general circulation model (AGCM) run under IO SST warming (Supplementary Fig. S8), which is forced by the observed SST, suggesting the importance of using a fully coupled model rather than an offline AGCM to investigate the atmospheric processes under the IO warming. In addition, anomalous easterly winds push warm surface water westward, increasing the sea surface height (SSH) in the IO (Fig. 4c). This increases the mixed-layer depth and enhances surface warming in the IO. The associated negative (positive) wind stress curl covers the northern (southern) IO (Supplementary Fig. S9a), which induces anomalous Ekman downwelling across the entire IO in the model. This deepens the thermocline over most of the IO (Supplementary Fig. S9b) and leads to large warming around the thermocline depth in the IO during the IPO warm phase (Fig. 4d). In summary, the warm TEP SSTs associated with an IPO warm phase induce positive SLP anomalies over the tropical western Pacific and IO and surface wind anomalies that weaken the tropical Walker circulation, reduce cloudiness, increase the SSH, and thus enhance the SSTs in the IO.

## Discussion and Conclusions

We realize that the average over a relatively small number (three) of ensemble runs used here may not completely remove internal variations. However, our analysis of the individual runs suggests that the ensemble mean of the three *All forcing* members, when combined with the 8-year low-pass filtering, can greatly reduce the decadal signal associated with the IPO (Supplementary Fig. S10). We also notice that the SST changes from the HadISST dataset over the IO are comparable to other datasets (Supplementary Fig. S11), with positive (negative) contributions to IO SSTs from the IPO mode during 1977–1998 (1947–1976) and far smaller contributions from the AMO mode. Thus we believe that the results presented here are robust (i.e., qualitatively insensitive to the choice of the SST datasets).

Based on the analyses above, the cold IPO phase since around 1999 should impose a negative anomaly and slow down its upward trend for the IO-averaged SST. This should contribute to the recent global warming hiatus by about 10%, although the IPO-induced cooling in the eastern pacific and other regions plays a bigger role^25^,^29^. We recognize that the IO and Pacific Ocean are in a coupled system in which the two can affect each other. For example, some studies have indicated an active role of the IO in affecting the Indo-Pacific climate during recent decades^11^. In particular, the enhanced tropical warming in the IO since 1950 relative to the Pacific Ocean favors stronger trade winds over the Pacific^30^, and the stronger trade winds are linked to the recent cooling in the eastern Pacific^31^. Thus, differential warming patterns in the tropical oceans may alter surface winds and thus tropical SSTs, including those over the TEP. However, climate models do not show enhanced warming over the IO compared with other tropical oceans in response to increased greenhouse gas (GHG) forcing^32^. This suggests that the observed warming trend difference since 1950 between the IO and Pacific may be due to the larger effect of the IPO on the Pacific warming trend than on the IO warming trend, because our result (Fig. 3c) and other studies^9^,^10^ suggest that the observed warming trend since 1950 in the IO is largely attributable to the historical external forcing with little effect from the IPO.

In summary, we found that the decadal variations in IO SSTs are closely linked to the IPO-induced variations in the TEP. Our results strongly suggest that decadal variations in the tropical IO and Pacific Ocean are tightly coupled and strongly influenced by the IPO.

### Data, Model Experiments and Methods

#### Observational and reanalysis data

Monthly SST data were obtained from the Hadley Centre Global Sea Ice and Sea Surface Temperature (HadISST) dataset^24^, National Oceanic and Atmospheric Administration Extended Reconstructed SST version 3 (ERSST_v3)^33^, Kaplan Extended SST version 2 (Kaplan_v2)^34^. Monthly SLP data were taken from Hadley Centre SLP version 2 (HadSLP2) dataset^35^, the 40-yr European Centre for Medium-Range Weather Forecasts (ECMWF) Re-Analysis (ERA-40)^36^. Data for 10 m winds were obtained from the Japanese 55-year Reanalysis (JRA-55)^37^ and ERA-40^36^.

#### Model and experiments

To examine the effects of the external forcing, internal variability and IPO on the IO SST, we designed and performed three sets of model experiments (Supplementary Table S1) using NCAR’s CESM1.2, a state-of-the-art fully coupled climate system model (Supplementary Table S2)^26^. The *All forcing* runs were forced by the historical radiative forcing for 1850–2005 and the Representative Concentration Pathway 4.5 (RCP4.5) for 2006–2012 based on CMIP5^27^. In the *All forcing* + *TEP SST* and *piControl* + *TEP SST* experiments, SSTs were prescribed to the model climatology plus observed anomalies in the TEP domain (15°S–15°N, 80°–180°W), along with the historical external forcing and fixed pre-industrial forcing, respectively. Supplementary Fig. S4 shows the TEP region where the monthly SST anomaly was fully prescribed as observed, while in a buffer zone (around and outside the outlined box in Supplementary Fig. S4 within five model grid boxes) the SST anomaly was blended with and relaxed to the modeled one. The oceans in other basins (including the IO) were fully coupled with the atmosphere.

As the internal variability is not synchronous across the individual realizations in a given set of experiments, we use their ensemble mean to suppress the effect of internal variability and highlight the externally forced signal. The results for the IPO mode from the individual runs support this method (Supplementary Fig. S10). A similar method to remove internal variability using a three-member ensemble mean has been used in previous studies^38^. Therefore, we can use the three-member ensemble mean of the *All forcing* runs to approximately represent the effects of external forcing on surface temperature averaged over a large domain^39^, which is close to the result from CMIP5 multi model mean, and also use the three-member ensemble mean of the *All forcing* + *TEP SST* runs to analyze the effects of both the external forcing and the TEP SSTs. The result of *piControl* + *TEP SST* run reflects the influences of the observed TEP SST, while the internal variability component of TEP SST effect can be obtained by the differences between the ensemble mean of *All forcing* + *TEP SST* runs and the ensemble mean of *All forcing* runs.

## Methods

To distinguish the individual effects of global warming, the IPO and AMO, an empirical orthogonal function (EOF) analysis^40^ of the 8-year low-pass filtered SST data from HadISST over global oceans during 1901–2011 was conducted. Based on their temporal and spatial characteristics, the first leading EOF mode represents the long-term global warming, and the second and third EOF modes depict the IPO and the AMO modes, respectively^23^,^41^. We compare the contributions of these three modes by reconstructing the SST anomalies represented by their corresponding EOF patterns and principal component (PC) time series^23^. Following Han *et al.*^11^, the dominant mode of the IO decadal variability was defined as the first leading EOF mode of the 8-year low-pass filtered SSTs over the IO (30°S–30°N, 40°–115°E), with the long-term linear trend removed. The 8-year low-pass filter using 21 points was done to remove the inter-annual variations using Lanczos filters^42^. To derive the IO’s contribution to the recent global warming hiatus (~10%), the following method is applied: a) Convert the IO-averaged anomalies from int_OBS in Fig. 3b to a global anomaly by multiplying it by the fraction of the IO area divided by the global area; b) Based on Dai *et al.*^25^, we can get the size of the decadal anomalies in global-averaged surface temperature during the same period after removing the forced trend; c) Compare the above two numbers can give a rough estimate of the contribution of the IO to the global warming hiatus since 1999.

## Additional Information

**How to cite this article**: Dong, L. *et al.* The Footprint of the Inter-decadal Pacific Oscillation in Indian Ocean Sea Surface Temperatures. *Sci. Rep.* **6**, 21251; doi: 10.1038/srep21251 (2016).

## Supplementary Material

Supplementary Information
