# Supplementary material for: The Footprint of the Inter-decadal Pacific Oscillation in Indian Ocean Sea Surface Temperatures
Source: Sci Rep. 2016 Feb 17;6:21251. doi: 10.1038/srep21251 (PMC4756665; doi:10.1038/srep21251)
Supplement: Supplementary Information [file srep21251-s1.doc]

The Footprint of the Inter-decadal Pacific Oscillation in Indian Ocean Sea Surface Temperatures

Lu Dong^1,2^, Tianjun Zhou^1,3*^, Aiguo Dai^4,5^, Fengfei Song^1,2^, Bo Wu^1^, Xiaolong Chen^1,2^

*1 State Key Laboratory of Numerical Modeling for Atmospheric Sciences and Geophysical Fluid Dynamics, Institute of Atmospheric Physics, Chinese Academy of Sciences, Beijing 100029, China*

*2 University of Chinese Academy of Sciences, Beijing 100049, China*

*3 Joint Center for Global Change Studies (JCGCS), Beijing 100875, China*

*4 Department of Atmospheric and Environmental Sciences, University at Albany, SUNY, Albany, New York, USA*

*5 National Center for Atmospheric Research (NCAR), Boulder, CO, USA*

Submitted to ***Scientific Reports***

**(Supplementary Information)**

**Table S1.** The experiments used in this study. The number denotes the ensemble members for each experiment. *TEP* stands for the tropical eastern Pacific Ocean (15°S–15°N, 80°–180°W).

| Experiment | Number (time span) |
| --- | --- |
| All forcing | 3 (1850–2012) |
| All forcing + TEP SST | 3 (1950–2012)  1 of the 3 (1871-2012) |
| piControl + TEP SST | 1 (1950–2012) |

**Table S2.** Basic information regarding CESM1.2^1^. Entries are as follows: GHG: greenhouse gas; AA: anthropogenic aerosol; BC: black carbon; OC: organic carbon; Ds: dust; Vl: volcanic; SS: sea salt; LU: land use; Sl: solar; SD: anthropogenic sulfate aerosol, accounting only for direct effects; MD: mineral dust; Oz = TO + SO: ozone = tropospheric and stratospheric ozone.

| Components | Resolutions | Forcing agents |
| --- | --- | --- |
| Atmosphere model: CAM5  Land model: CLM4  Sea ice model: CICE4  Ocean model: POP2  Land ice model: Glimmer_CISM  Coupler: CPL7 | CAM5: 0.9° latitude × 1.25° longitude, 27 vertical levels  CICE4 & POP2: Nominal 1° (1.125° in longitude, 0.27–0.64° variable in latitude), 60 vertical levels | GHG, AA, Sl, Vl, SS, Ds, SD, BC, MD, OC, Oz, LU  Aerosol component type: Semi-interactive  External forcing estimates in historical run^2^ and RCP run^3^ |


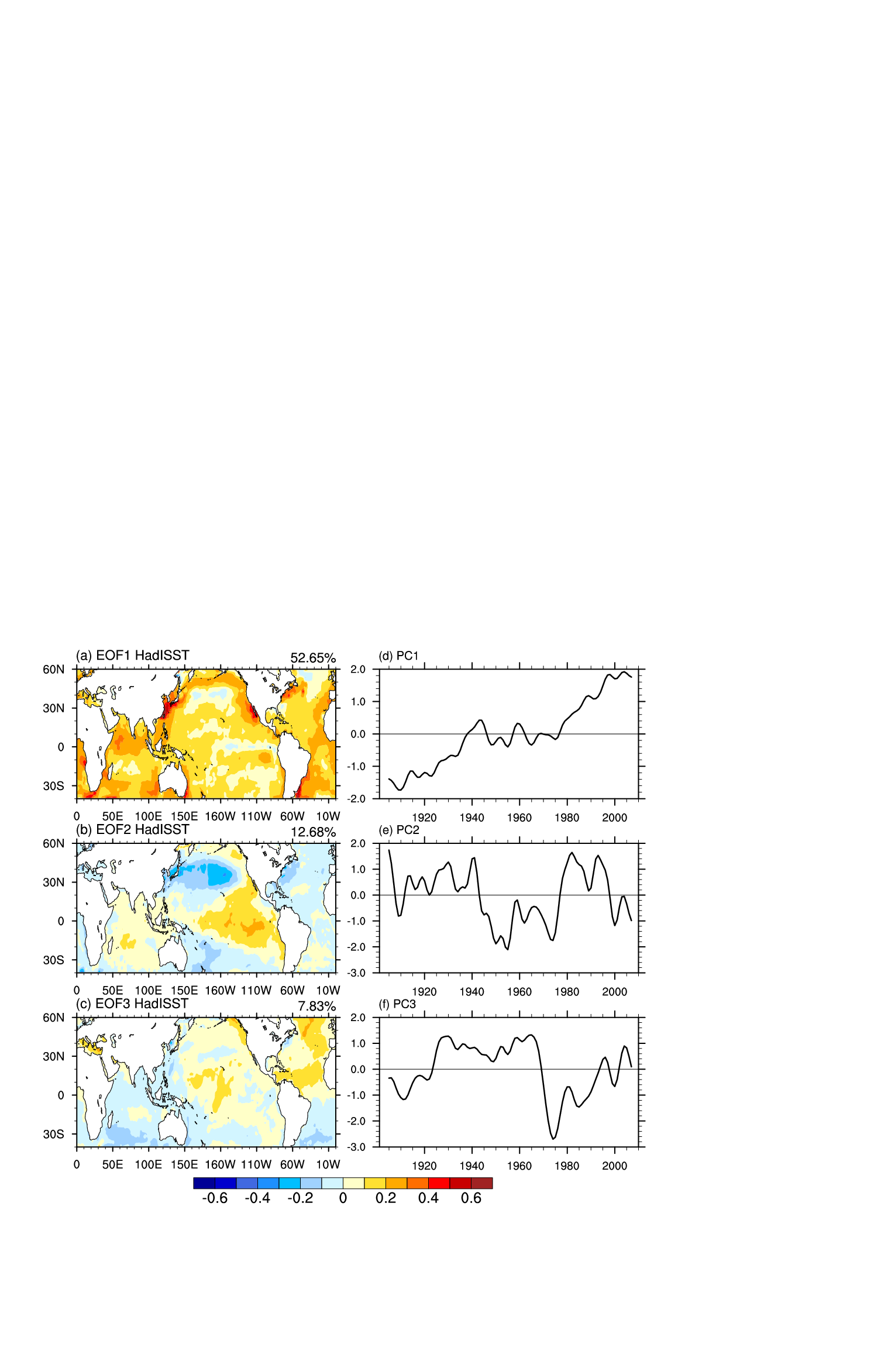


**Fig. S1.** The first three leading EOF patterns and standardized PC time series of 8-year low-pass filtered SSTs from HadISST. (**a, d**) The first EOF mode, (**b, e**) the second EOF mode, (**c, f**) the third EOF mode. This plot was created by NCAR Command Language^4^.


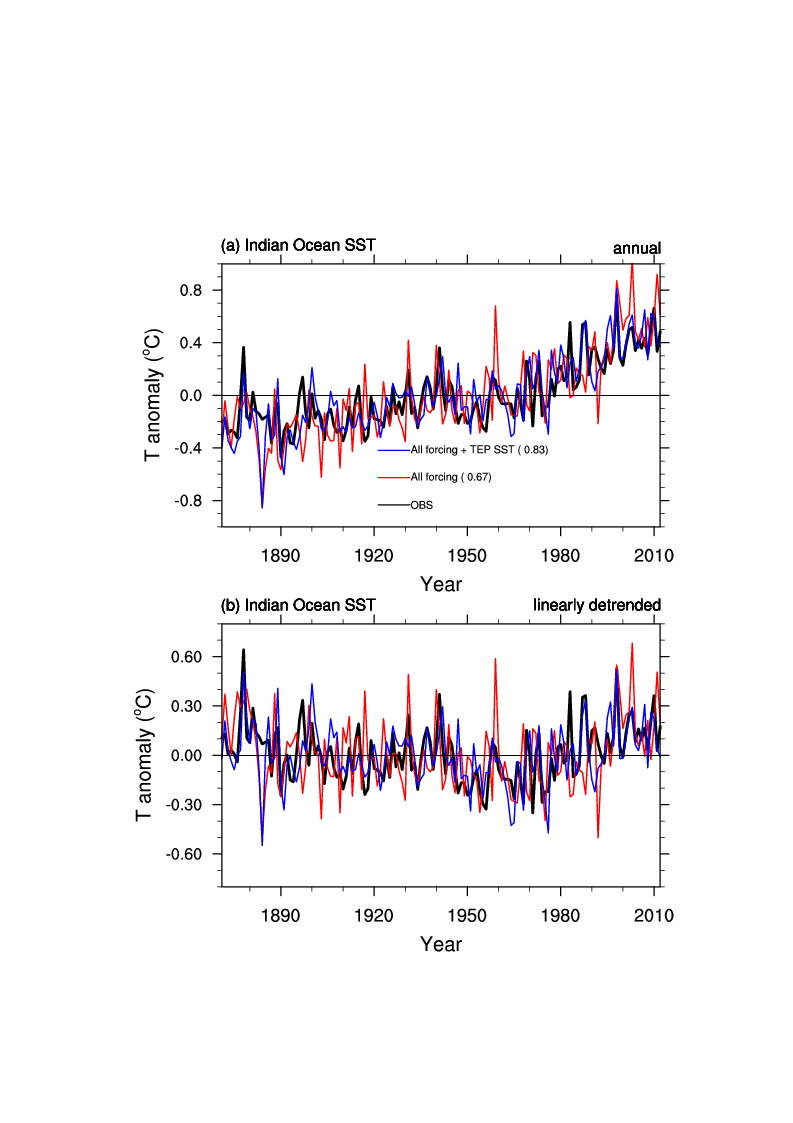


**Fig. S2.** (**a**) Time series of the annual mean SST anomaly (^o^C) averaged in the Indian Ocean (20°S–20°N, 40°E–120°E) for HadISST (black), the *All forcing* run (red) and the *All forcing + TEP SST* run (blue) during 1871–2012. The values in the brackets are the correlation coefficients with the HadISST curve. Plot (**b**) as the results after the linear trends are removed. This plot was created by NCAR Command Language^4^.


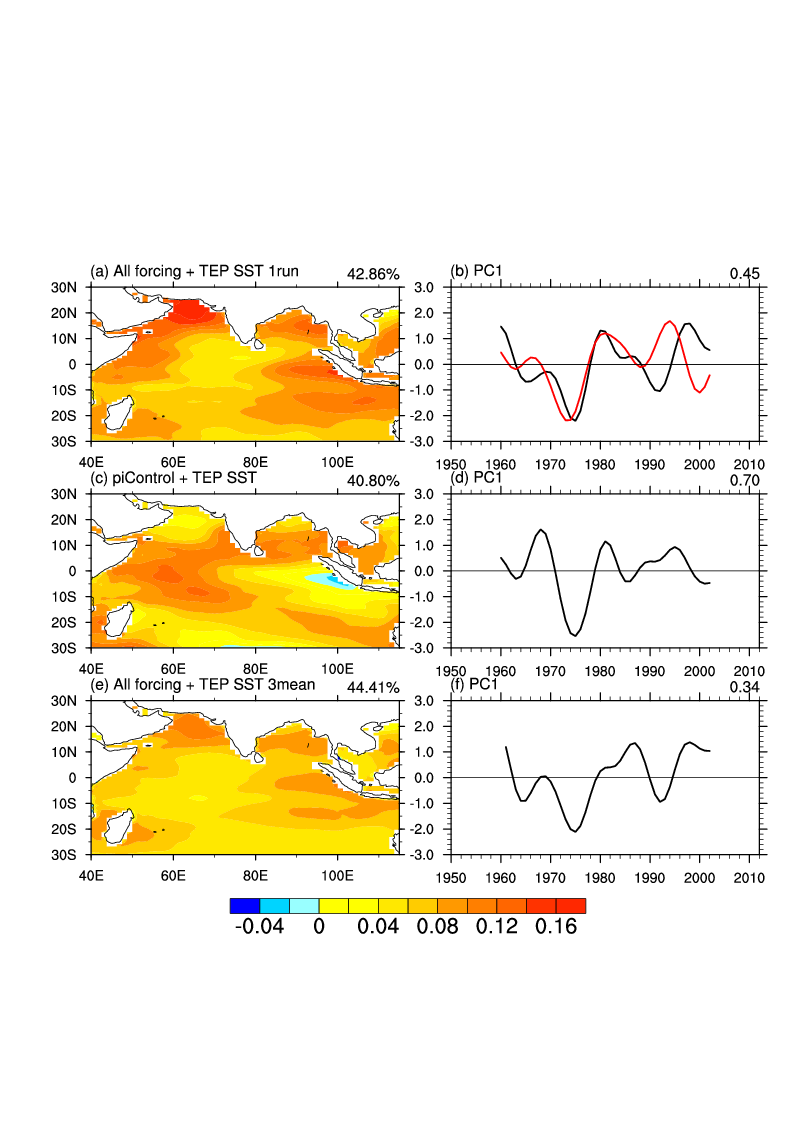


**Fig. S3.** The leading EOF pattern and standardized PC of 8-year low-pass filtered SSTs in the Indian Ocean for (**a**–**b**) the *All forcing + TEP SST* run, (**c**–**d**) the *piControl+TEP SST* run, and (**e**–**f**) the ensemble mean of the three *All forcing + TEP SST* runs during 1950–2012. The red line in (b) represent the observed IPO index. The values given in the top-right in (**a**, **c**, **e**) denote the explained percentage variance by the EOF mode, and those in (**b**, **d**, **f**) denote the correlation with IPO. This plot was created by NCAR Command Language^4^.


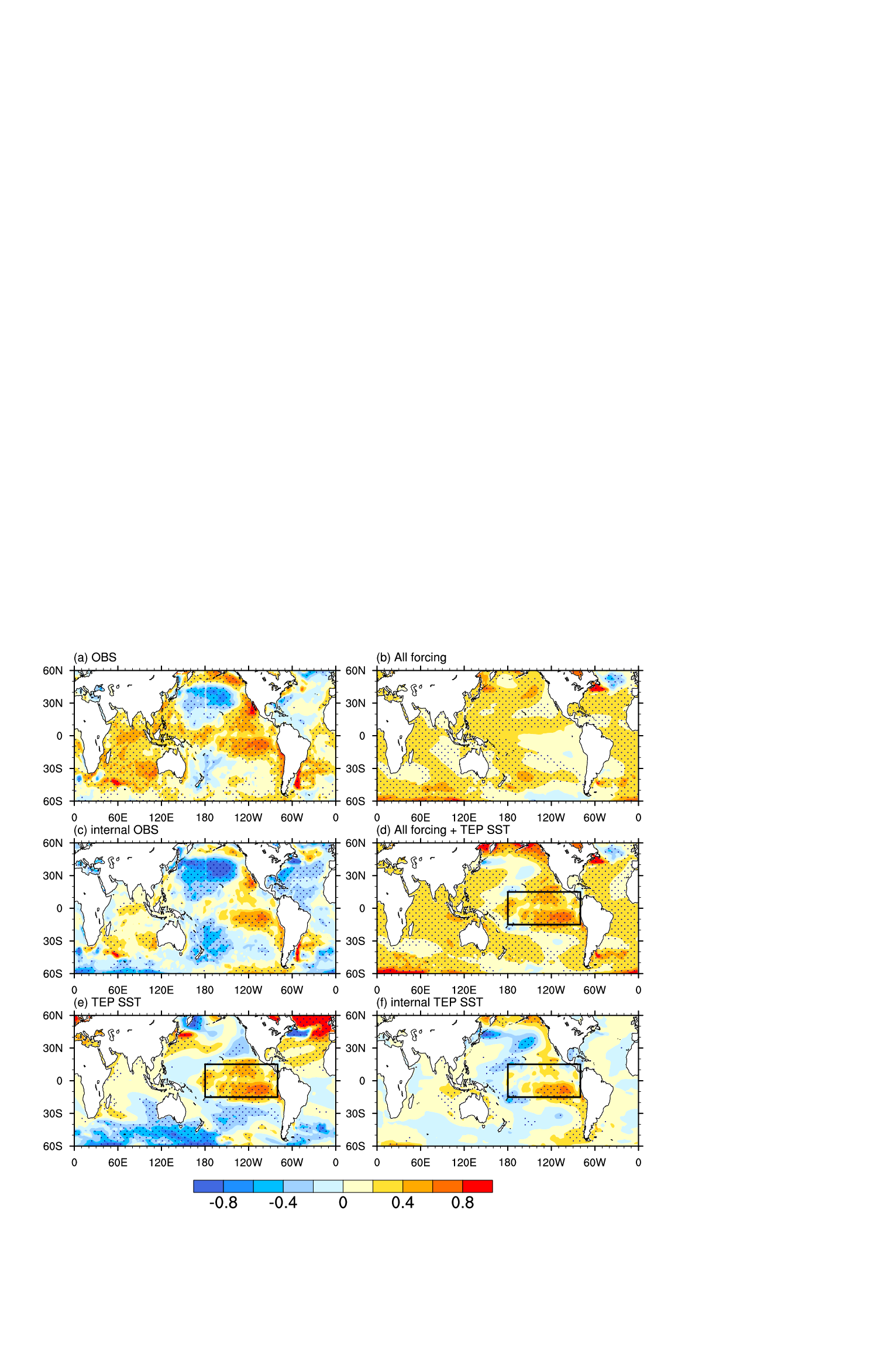


**Fig. S4.** The 1977-1998 minus 1951-1976 decadal change patterns of SSTs (^o^C) from (**a**) HadISST, (**b**) the ensemble mean of the three *All forcing* runs, (**c**) the internal variability in HadISST derived using (**a**) minus (**b**), (**d**) the ensemble mean of the three *All forcing + TEP SST* runs, (**e**) the *piControl + TEP SST* run, and (**f**) due to the internal TEP SST forcing derived using (**d**) minus (**b**). The box outlined by the black line represent the TEP domain where SST anomalies were prescribed as observed in all the "*+TEP SST*" runs. The dotted areas are statistically signiﬁcant at the 5% level based on a Student’s *t*-test. This plot was created by NCAR Command Language^4^.


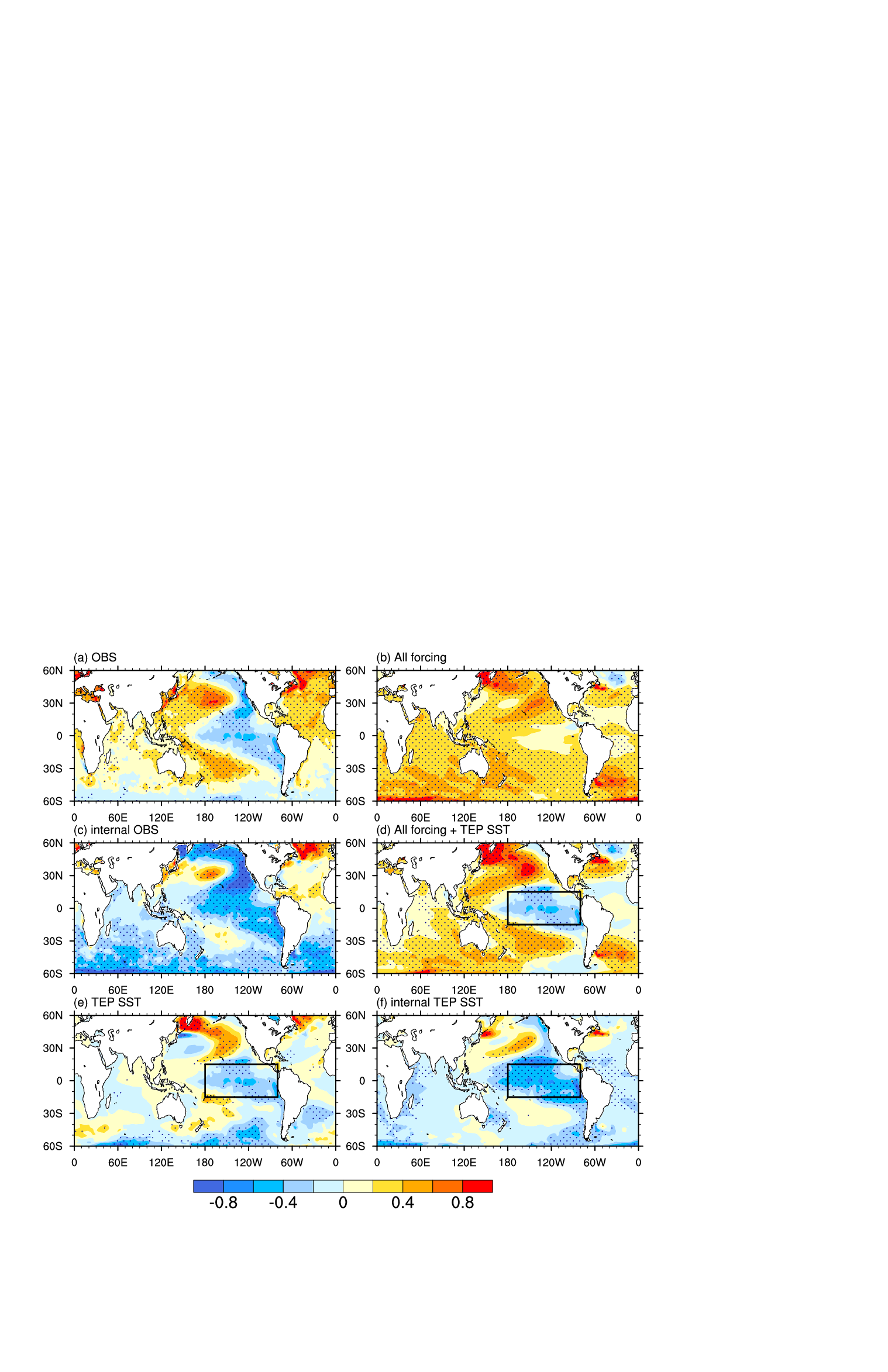


**Fig. S5.** As in Figure S4, but for the 1999-2012 minus 1977-1998 decadal change patterns of SSTs. This plot was created by NCAR Command Language^4^.


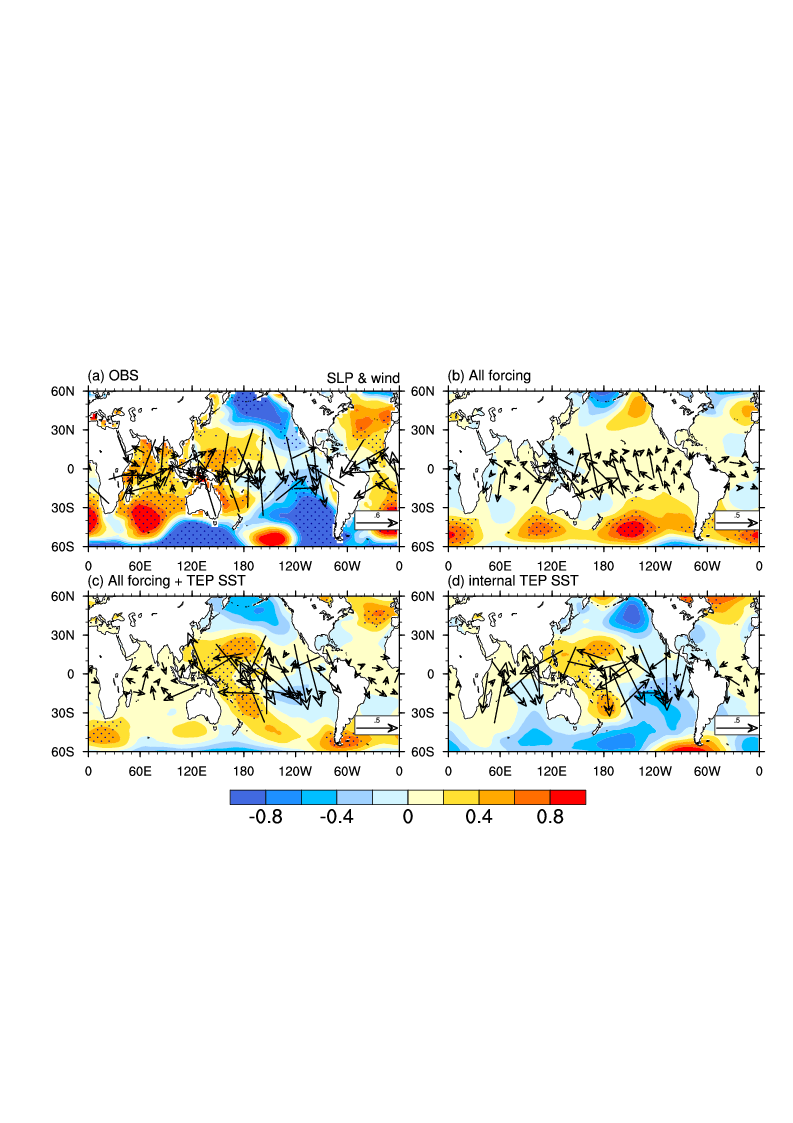


**Fig. S6.** The 1977-1998 minus 1951-1976 decadal change patterns of sea level pressure and surface wind vectors from (**a**) ERA40 (1977-1998 minus 1958-1976), (**b**) the ensemble mean of the three *All forcing* runs, (**c**) the ensemble mean of the three *All forcing + TEP SST* runs, and (**d**) due to the internal TEP SST forcing derived using (**c**) minus (**b**). The dotted areas are statistically signiﬁcant at the 5% level based on a Student’s *t*-test. This plot was created by NCAR Command Language^4^.


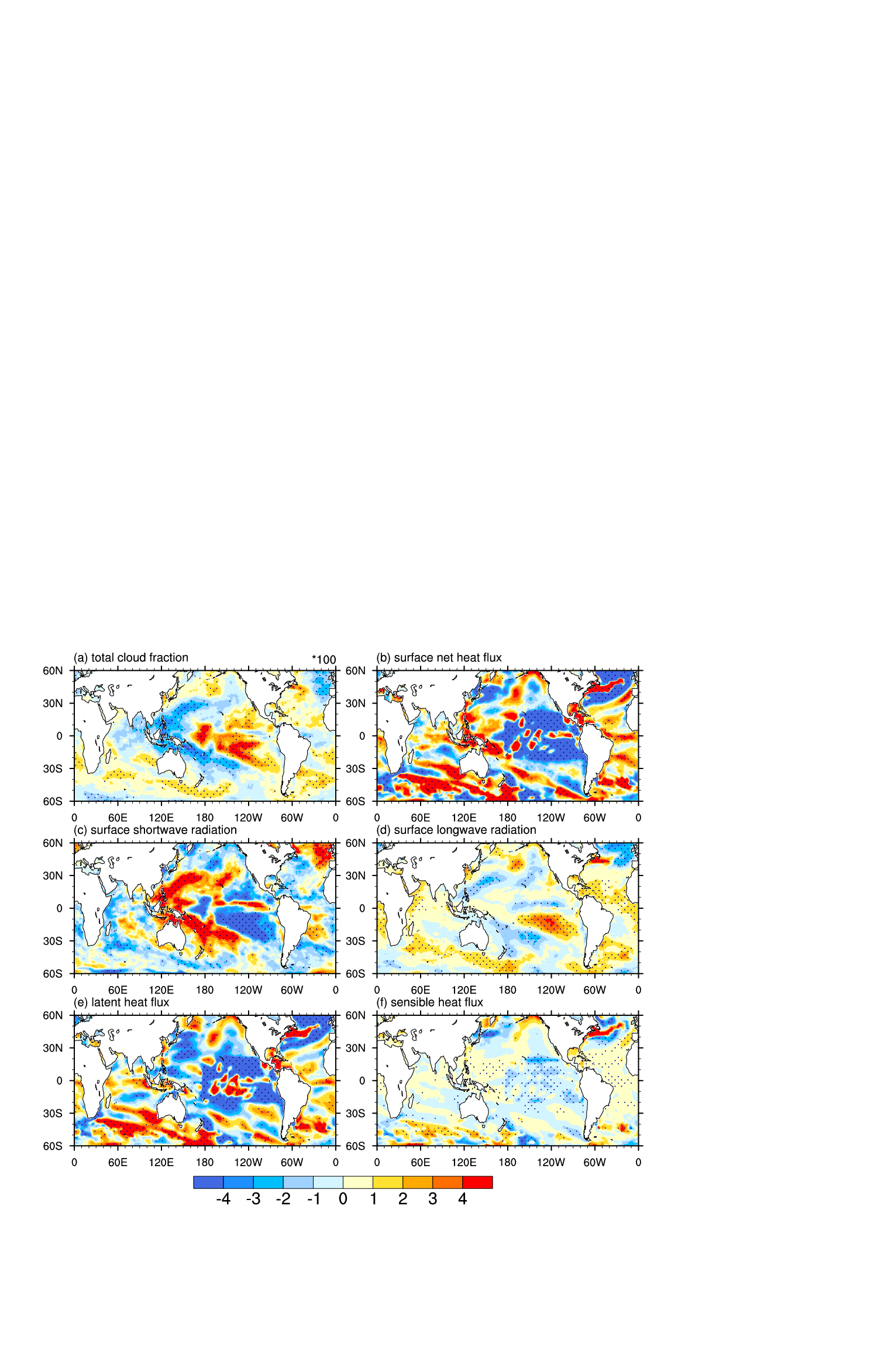


**Fig. S7.** The 1977-1998 minus 1951-1976 decadal change patterns of (**a**) total cloud cover (in %), (**b**) surface net energy flux, (**c**) surface net shortwave radiation, (**d**) surface net longwave radiation, (**e**) latent heat flux, and (**f**) sensible heat flux from the *piControl + TEP SST* run. Values in (**b**–**f**) are in W m^-2^ and positive downward. The dotted areas are statistically signiﬁcant at the 5% level based on a Student’s *t*-test. This plot was created by NCAR Command Language^4^.

**Fig. S8.** (a) The trends of total cloud fraction during 1979-2010 from the CCSM4 AMIP (AGCM) run (Units: decade^-1^). (b) The 1977-1998 minus 1951-1976 decadal change patterns of total cloud fraction from the CanAM4 AMIP run. The dotted areas are statistically signiﬁcant at the 5% level based on a Student’s *t*-test. This plot was created by NCAR Command Language^4^.

**
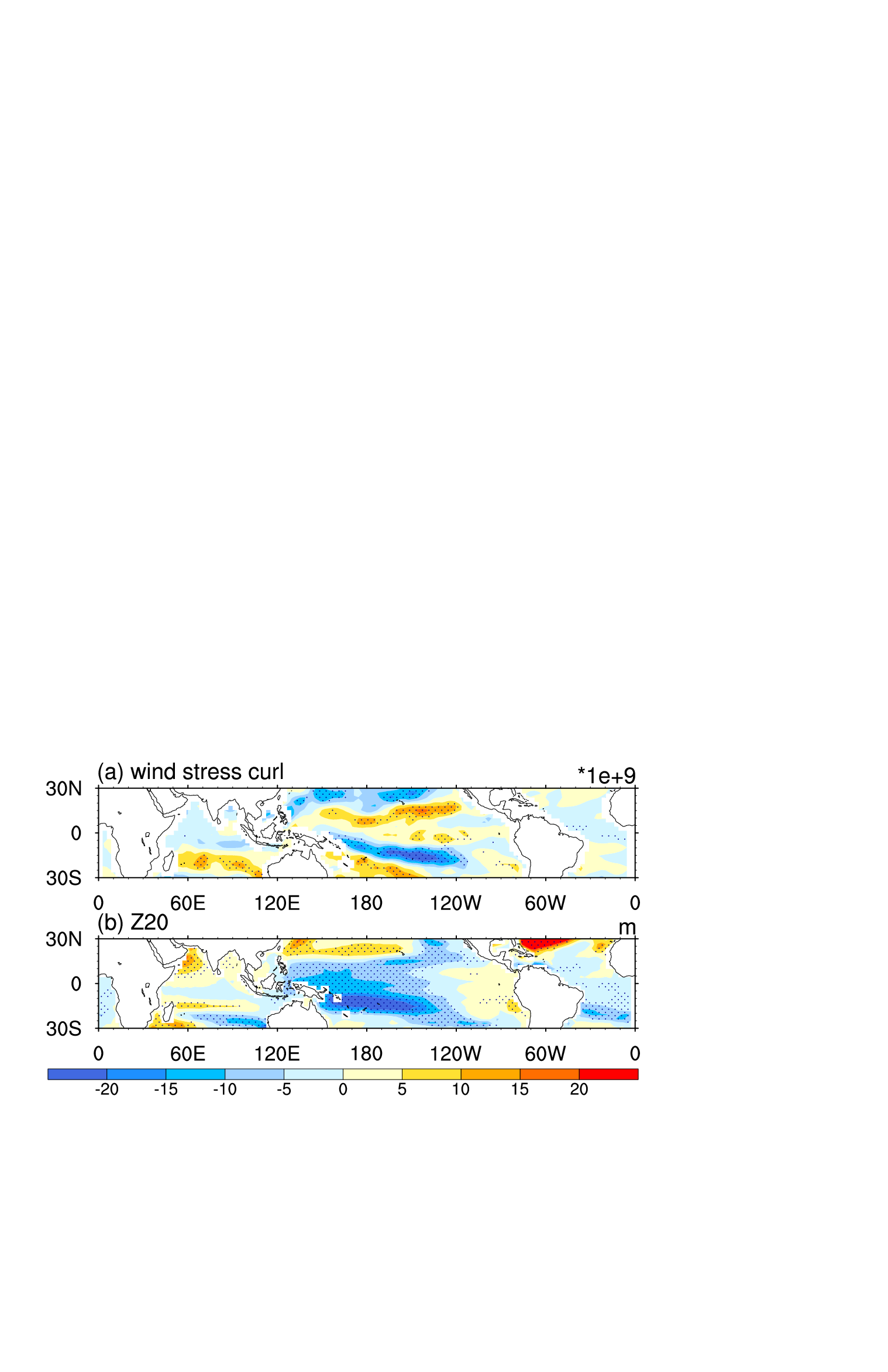
**

**Fig. S9.** The 1977-1998 minus 1951-1976 decadal change patterns of (**a**) wind stress curl (N/m^3^) and (**b**) 20°C isotherm depth (m) from the *piControl + TEP SST* run. The dotted areas are statistically signiﬁcant at the 5% level based on a Student’s *t*-test. This plot was created by NCAR Command Language^4^.

**
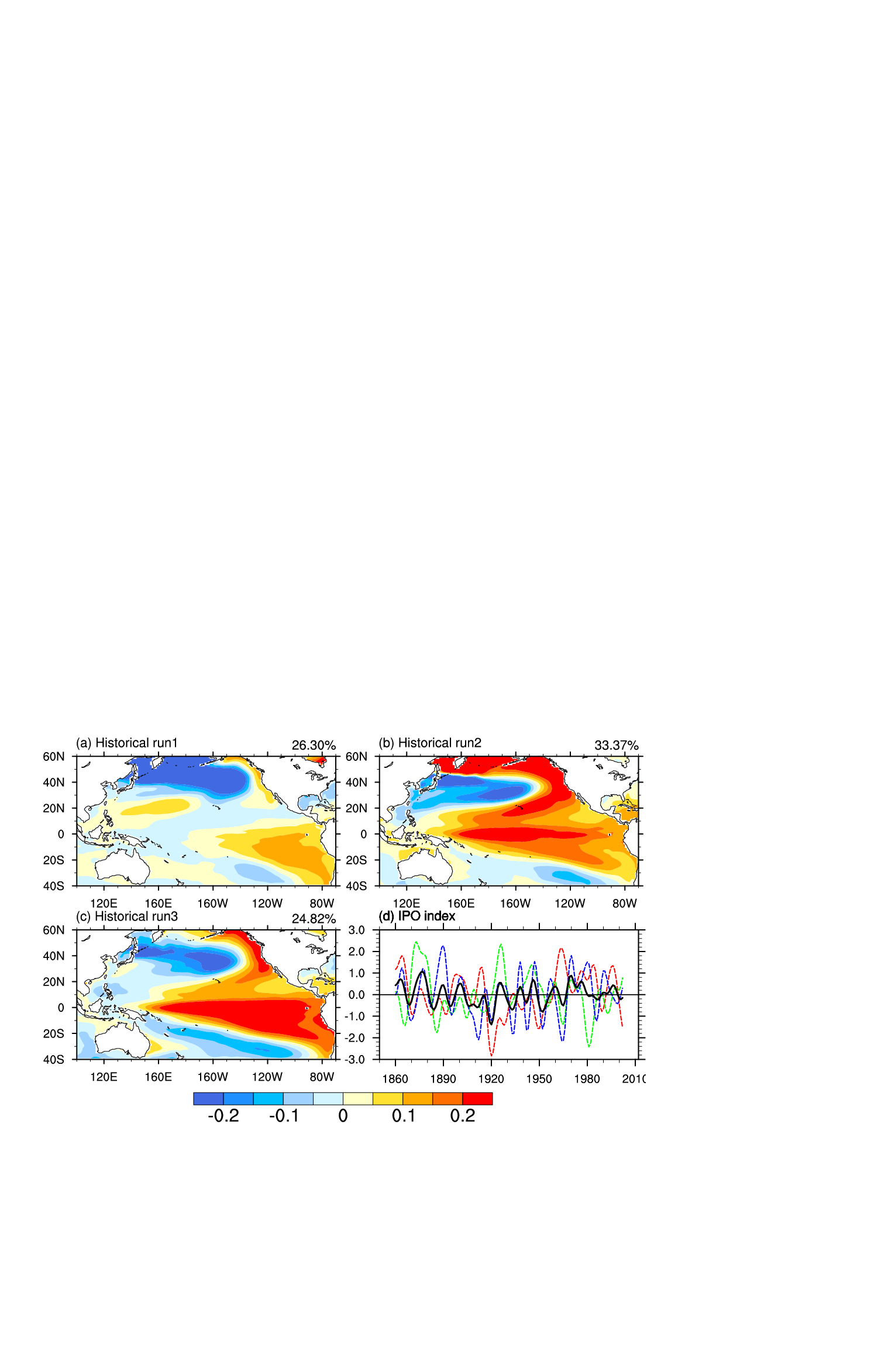
**

**Fig. S10.** The (**a**–**c**) spatial pattern and (**d**) time series of the IPO mode in each of the three *All forcing* runs during 1850–2012. The red, green, blue and black lines in (**d**) are for run #1, 2 and 3, and the ensemble mean of the three runs, respectively. The IPO is defined as the first EOF mode of the 8-year low-pass filtered and linearly detrended SSTs over the Pacific Ocean (40°S–60°N, 100°E–70 °W). This plot was created by NCAR Command Language^4^.


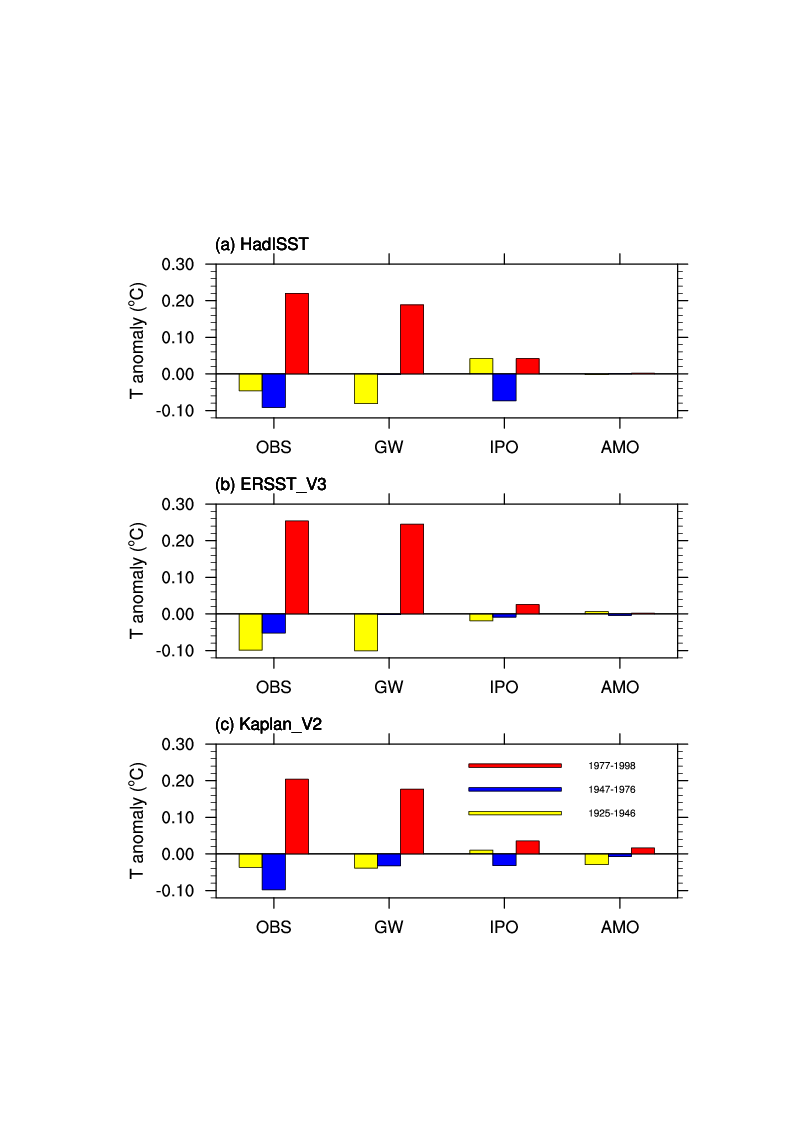


**Fig. S11.** Epoch mean of the Indian Ocean SST anomalies during the warm IPO phase (1925–1946, yellow bars), the cold IPO phase (1947–1976, blue bars), and the warm IPO phase (1977–1998, red bars) from (**a**) HadISST, (**b**) ERSST_V3, (**c**) Kaplan_V2. This plot was created by NCAR Command Language^4^.

**References for SI**

1. Hurrell, J. et al. The Community Earth System Model: A Framework for Collaborative Research. *Bull. Amer. Meteor. Soc.* **94**, 1339–1360 (2013).
2. Lamarque, J. F. et al. Historical (1850–2000) gridded anthropogenic and biomass burning emissions of reactive gases and aerosols: Methodology and application. *Atmos. Chem. Phys.* **10**, 7017–7039 (2010).
3. van Vuuren, D. P. et al. Stabilizing greenhouse gas concentrations at low levels: An assessment of reduction strategies and costs. *Climatic Change* **81**, 119–159 (2007).
4. The NCAR Command Language (Version 6.1.2) [Software]. (2013). Boulder, Colorado: UCAR/NCAR/CISL/VETS. http://dx.doi.org/10.5065/D6WD3XH5.
